# Educational attainment is associated with unconditional helping behaviour

**DOI:** 10.1017/ehs.2019.16

**Published:** 2019-12-11

**Authors:** Grace Westlake, David Coall, Cyril C. Grueter

**Affiliations:** 1 School of Human Sciences, The University of Western Australia, M309, LB 5005, Perth, WA 6001, Australia; 2 School of Medical and Health Sciences, Edith Cowan University, Joondalup, WA 6027, Australia; 3 School of Medicine, The University of Western Australia, Crawley, WA 6009, Australia; 4 Centre for Evolutionary Biology, School of Biological Sciences, The University of Western Australia, Perth, WA 6001, Australia

**Keywords:** Altruism, prosocial behaviour, lost letter experiment, socio-economic status, education

## Abstract

Altruism is a universal human trait, but little is known about its within-population variation. Socio-economic status (SES) has been found to positively impact altruism, but the *specific* socio-economic variables behind this relationship have remained elusive. This study aimed to determine which facets of SES predict altruism using a lost letter paradigm and a novel lost letter method. Six hundred letters (half dropped on the pavement, half sent to residential addresses) were distributed in 20 suburbs of Perth (Australia) differing in socio-economic variables. Letters distributed in high-SES neighbourhoods were more likely to be returned than letters distributed in low-SES neighbourhoods. Educational attainment and occupation status were the specific socio-economic variables underlying this association, while economic resources and crime rate were not associated with the likelihood of a letter being returned. These results suggest that altruism blossoms in neighbourhoods that are populated with highly educated individuals working in high-status jobs. The relationship between education and prosocial inclinations may be mediated by cognitive ability, self-control and high levels of socialization. Having experienced sustained exposure to norm-abiding models, more educated people may also be better at internalizing cultural norms of helping behaviour, thus creating a more altruistic environment where they reside.

## Graphical abstract

**Figure:**
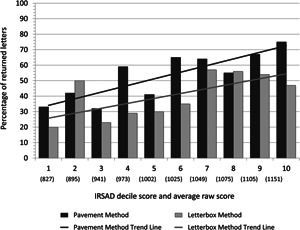

**Media summary**: A field experiment suggest that altruism blossoms in neighbourhoods populated with highly educated individuals working in high-status jobs.

## Introduction

Cooperation – a behaviour that provides a benefit to another individual – is a human universal. Cooperative behaviour between relatives is typically governed by kin selection, which suggests that individuals can increase their inclusive fitness by helping genetic relatives (Hamilton 1964). Cooperation involving non-relatives (and sometimes relatives; Allen-Arave *et al*. 2008) can be explained by direct reciprocity, which involves exchanges of favours between individuals who are expected to have future encounters (Trivers 1971; Axelrod and Hamilton 1981). Furthermore, individuals can help others in order to enhance their own reputation through indirect reciprocity (Alexander 1987; Nowak and Sigmund 1998). However, these reciprocity explanations require either the recipient or a witness to be aware of the actor's identity. Some altruistic acts (i.e. behaviours that are costly to the actor and beneficial to the recipient) are anonymous whereby the recipient of the act is not aware of the actor's identity and no third party witnesses the act. These types of anonymous altruistic acts reflect unconditional or genuine altruism, which is altruism without an expectation of future reciprocation and without reputational benefits.

Explanations of prosocial behaviour in large groups of unrelated individuals fall into two broad categories. First, the ‘mismatch’ hypothesis posits that helping behaviour in present-day large-scale societies is the result of psychological dispositions for cooperation that evolved mainly in the context of repeated interactions with known individuals in ancestral environments in which the actor would have obtained benefits through any of the aforementioned theories (kin selection, direct and indirect reciprocity; e.g. Burnham and Johnson 2005). However, this hypothesis has been challenged on multiple grounds. First, even in past environments, ephemeral interactions with strangers probably took place. Further, this hypothesis makes the questionable assumption that humans lack the ability to adjust behaviour in light of knowledge about the probability of future payoffs from an interaction (Chudek *et al*. 2013; Fehr and Henrich 2003; Raihani and Bshary 2015). Second, cultural group selection suggests that groups whose members engage in individually costly, cooperative, altruistic behaviours may have a competitive advantage over other groups whose members do not cooperate, which can lead to the emergence of locally stable equilibria of prosocial norms (Gintis 2000; Henrich 2004; Richerson *et al*. 2003, 2016; Chudek and Henrich 2011; but see Mollemen *et al*. 2013; Raihani and Bshary 2015). The evolution of cooperative cultural norms within populations is facilitated by an inclination for conformity-based social learning (Henrich and Boyd 2001; but see André and Morin 2011).

Altruistic behaviours do not manifest in human populations at uniform levels (Levine *et al*. 2001; Richerson *et al*. 2016). The prevalence of altruism may thus be subject to contextual differences, meaning that it may have different fitness benefits in different contexts. Cross-cultural variation in cooperative tendencies has been shown to be present in many small-scale societies (Henrich *et al*. 2005, 2006, 2010), as well as industrialized societies (Hermann *et al*. 2008; Roth *et al*. 1991). These population-level differences may be mediated by culturally inherited cooperative norms (Henrich 2004; Henrich *et al*. 2005, 2006; Richerson *et al*. 2016). Lamba and Mace (2011) have suggested that differences in cultural norms can be explained by demographic and ecological differences such as population size and age structures. This suggests that contextual differences can create variation in altruistic behaviour *between* different populations. However, little is known about the degree to which these factors may affect variation *within* populations. Cultural group selection suggests that social norms may result in relatively uniform altruistic behaviour within a group. However, there is much evidence to suggest that groups do not always exhibit uniform behaviours, and that within-population variation in altruistic behaviour does exist (Levine *et al*. 2001; Richerson *et al*. 2016).

One method that has been used to investigate altruistic behaviour is the lost letter experiment (Milgram *et al*. 1965). The lost letter experiment involves scattering addressed, stamped and sealed envelopes throughout an area, so that each letter looks as though someone accidentally dropped it while on the way to post it. These letters are then naturalistically encountered by random individuals in the area. If choosing to act altruistically, individuals can pick up the letter and post it through a post box, or if choosing to not act altruistically, individuals can ignore it. The rate of letters that are returned is used as a proxy for altruism in a geographical area; a high return rate indicates a high willingness of individuals to be altruistic, while a low return rate indicates a low willingness. The lost letter experiment primarily addresses unconditional altruism, because it is a largely anonymous altruistic act that cannot be reciprocated. The naturalistic nature and resulting high ecological validity make the lost letter experiment a useful tool to investigate heterogeneity in prosocial behaviour on a geographic and socioeconomic scale. Despite the relatively widespread use of the lost letter method to examine human behaviour, it has some limitations such as non-residents encountering letters and the effects of differing pedestrian foot traffic.

It has been widely shown that the letter return rate is positively correlated with increasing socio-economic status (SES); letters distributed in high-SES neighbourhoods are returned at a higher rate than letters distributed in low-SES neighbourhoods (Brown and Reed 1982; Chang *et al*. 2016; Grueter *et al*. 2016; Holland *et al*. 2012; Nettle *et al*. 2011; Silva and Mace 2014). However, the exact mechanisms behind this effect are unknown. Socio-economic status is a multi-dimensional measure that is composed of many different variables such as economic resources, educational attainment, occupation status, employment status, crime and family structure variables such as the marital status of parents. It is unclear which of the many components of SES affect the tendencies of individuals to act altruistically.

One possible dimension of SES that may affect altruism is economic resources. Economically deprived individuals may not have stable access to basic needs such as food and housing and may thus be primarily focused on meeting these immediate needs, leaving them with less time and energy to spend helping others. Conversely, individuals with greater economic resources may have abundant access to their basic needs, leaving them with reserve time and energy to spare for altruistic behaviour (Holland *et al*. 2012).

Alternative mechanisms through which SES may affect altruism are educational attainment and occupation status. Considering that socialization is an important component of school curricula and facilitates interactions with strangers (Glaeser *et al*. 2007), one could argue that higher levels of helping among the more educated may also reflect better social skills. Individuals with a higher educational attainment may also have a broader arsenal of cognitive abilities that may influence their prosociality. Jones (2008) found that cooperation in Prisoner's Dilemma games among university students increased with the universities’ average SAT score, which suggests that more educated and intelligent individuals may have a greater willingness or ability to cooperate. This was reinforced by Segal and Hershberger (1999), who administered IQ tests and Prisoner's Dilemma games to identical and fraternal twins. They found that pairs with a higher combined IQ were more likely to cooperate with each other. One explanation for these findings is that intelligent individuals may be more patient (Frederick 2005; Warner and Pleeter 2001). Curry *et al*. (2008) found that patient individuals were more cooperative in a series of economic games. They suggested that their preference for future over immediate rewards enabled them to engage successfully in long-term, reciprocal altruism. Additionally, using a cultural group selection perspective, education can be construed as the engine of a process whereby prosocial norms become internalized in a culturally prescribed social group. The upshot is the proliferation of positive attitudes towards helping behaviour among the educated.

Another mechanism by which SES may influence altruism is through environmental stability. Low-SES individuals may lack environmental stability owing to factors such as increased crime and poor healthcare. In these harsher and more unpredictable environments distant rewards are discounted, as they may never be attained, and behaviour is biased towards impulsivity, reward seeking and risk taking (Chisholm 1999; Coall *et al*. 2012). With shortened time horizons, the willingness of individuals to engage in behaviours without immediate benefits decreases (Holland *et al*. 2012; Maskin and Fudenberg 1986). This may cause low-SES individuals to be disinterested in long-term cooperation without immediate reciprocation. Furthermore, higher crime rates may decrease individuals’ trust of others, leading them to behave less altruistically (Alesina and Ferrara 2002). Trust is essential for altruism because individuals must be willing to accept vulnerability in order to cooperate with others (Piff *et al*. 2010).

Low-SES individuals have also been found to have a lower sense of control over their environments, exhibiting attitudes of helplessness about their socio-economic position (Gallo *et al*. 2005; Johnson and Krueger 2006; Kraus *et al*. 2012). They may believe that there is no benefit to behaving altruistically as it will not change their environment; no matter how they act, people will treat them the same. Therefore, there is no sense in wasting energy being altruistic. Conversely, high-SES individuals with a greater sense of control over their environment may have confidence that there are long-term reciprocal benefits of engaging altruistically. There is much that we do not know about the relationship between SES and altruism. The dimensions of SES need to be teased apart to establish which specific elements contribute towards these relationships. This will then enable us to focus on the mechanisms by which these elements can affect altruistic tendencies.

Using the lost letter technique, we analysed the association between a variety of area-level socio-economic variables (in particular economic resources, education and occupation and crime rate) and willingness to help a stranger. We also implemented a novel, modified lost letter experiment to rectify some limitations inherent in the traditional lost letter experiment.

## Methods

### Data collection

A total of 600 letters were distributed among 20 different suburbs of Perth, Western Australia (30 letters per suburb). Suburbs were selected based on a number of selection criteria such as being greater than three-quarters suburban, free from any rural qualities and of sufficient size to distribute 30 letters.

Twenty suburbs were selected to exhibit a range of different SES characteristics across economic resources, education and occupation, and crime rates. Suburbs were also selected such that these factors were not strongly correlated. Table S1 (in the Supplementary Information) lists all 20 suburbs selected and their relevant socio-economic statistics.

Within Perth, 300 letters were distributed using the original lost letter design, called the ‘pavement method’. A further 300 letters were distributed using a novel, refined method, called the ‘letterbox method’ (see below). In each of the 20 suburbs, 15 letters were distributed via the pavement method. Letters were stamped, sealed, and addressed to a residential address. Envelopes contained a note to reduce suspicion in case they were opened, and were coded so that their exact drop location could be identified. Letters were placed address-side up on pedestrian paths at evenly distributed, pre-determined locations within each suburb. Letters were distributed on Friday evenings to avoid postmen encountering the letters on weekdays and in good weather to ensure pedestrians would come across them.

In each of the 20 suburbs, 15 letters were also distributed via the new, modified lost letter design (the letterbox method). Letters were addressed and delivered to the letter boxes of residential houses, but featured a false name of someone who did not live there. Letters were designed to appear as though the sender had accidentally used the incorrect address of the recipient or made an error when writing it. Letters featured a sender address on the back of the letter so that if choosing to act altruistically, recipients of the letter could return the letter to the sender. Letterbox letters featured the same content as the pavement letters, and their distribution locations (addresses they were sent to) were selected to be evenly distributed across each suburb.

### Measures

Geographical area-level SES was measured using the Australian Bureau of Statistics (ABS) Index of Relative Socio-economic Advantage and Disadvantage (IRSAD; Australian Bureau of Statistics 2013, 2014a). This index consists of two sub-measures: Index of Economic Resources (IER; Australian Bureau of Statistics 2013, 2014b) and Index of Education and Occupation (IEO; Australian Bureau of Statistics 2013, 2014c), which were both used to measure different socio-economic dimensions. IRSAD aggregates the factors indicative of disadvantage (such as low income, level of education and private dwellings with no internet connection) and advantage (including annual income in 9th and 10th deciles and employed people classified as professionals). IER aggregates the factors indicative of disadvantage (such as low income, occupied dwellings with no cards and occupied dwellings paying rent less than AUD215 per week) and advantage (occupied dwellings with four or more bedrooms, occupied dwellings paying a mortgage greater than AUD2800 per month and people with annual household income greater than AUD78,000). IEO aggregates the factors indicative of disadvantage (such as the percentage of people who work in low-skill occupations, percentage of people who are unemployed and the percentage of people aged 15 and over who have no educational attainment) and advantage (percentage of employed people in a high-skill job and percentage of people aged 15 years and over at a university).These indices were obtained for each suburb, and also for smaller areas within each suburb called statistical areas level 1 (SA1; Australian Bureau of Statistics 2011) to test for within-suburb variation.

Crime rate was measured as the total number of crimes per 100,000 people using statistics of crime provided by the Western Australia Police Force from April 2015 to March 2016. Crime data were not available for areas smaller than a suburb, so the crime statistic was the same for the suburb-level and SA1-level analyses.

### Ethics

Ethics was granted by the Human Research Ethics Committee at The University of Western Australia in accordance with the National Statement on Ethical Conduct in Human Research (Reference number RA/4/1/8257).

### Data analysis

To determine which explanatory variable best predicted whether a letter would be retuned or not, a generalized linear mixed model (GLMM) with binomial error structure and logit link function (multilevel logistic regression) was run with the following fixed effects: letter distribution method (whether the letter was distributed via the pavement or letterbox method), post boxes (the number of post boxes in a suburb), crime rate and IRSAD. The number of post boxes was included to control for the ease of returning a letter across different suburbs. This was determined using an online post box map locator (Australia Post 2016). The suburb itself was included as a random effect to account for random unmeasured effects in each suburb. All data were analysed from both the pavement and letterbox methods. The main model was run twice, once using suburb-level data and once using SA1-level data (Table S2). This was done to determine the relative significance of each data level, and which level should be retained for subsequent analyses. The results were very similar for both data levels, so SA1 data were retained for the rest of the analysis because they provide more specific information for smaller areas.

The effect of SES on letter return rate was explored further by running two additional models with the sub-measures of IRSAD: IER and IEO. The above model was repeated twice, once by replacing the fixed-effect IRSAD with IER, and once by replacing it with IEO. Models with IRSAD, IER and IEO had to be run separately owing to their shared variables and high degree of collinearity (Pearson correlation coefficient for IRSAD and IEO, 0.86; IRSAD and IER, 0.93). Raw IRSAD, IER and IEO variables were used for analysis (not decile values).

GLMMs assume that fixed effects are not collinear. Therefore, as a diagnostic test, collinearity between crime and all other explanatory variables was examined using Pearson correlations (Dormann *et al*. 2013). None of the correlation coefficients were over 0.6 (crime and IRSAD, *r* = −0.55; crime and IER, *r* = −0.56; crime and IEO, *r* = −0.43). GLMMs also assume that there are significantly more cases/data points than estimated parameters (Harrison *et al*. 2018), and that there are no influential cases. Both of these assumptions were checked and satisfied. Before fitting the models, we *z*-transformed all quantitative predictor variables to a mean of 0 and a standard deviation of 1 to achieve comparable estimates and to increase the likelihood of model convergence (Schielzeth 2010). To check the overall significance of the combined set of predictor variables, we ran likelihood ratio tests comparing each full model with a respective null model containing only the intercept and random effects (Dobson 2002; Forstmeier and Schielzeth 2011). None of the two-way interactions between any of the SES variables and crime and post boxes, respectively, were significant; we thus recalculated the models without the interactions. All GLMMs were computed with the lme4 package (Bates *et al*. 2015) in R (R Core Team 2014) version 3.3.1. Wald confidence intervals were computed using the function ‘confint’.

## Results

**Figure 1.**
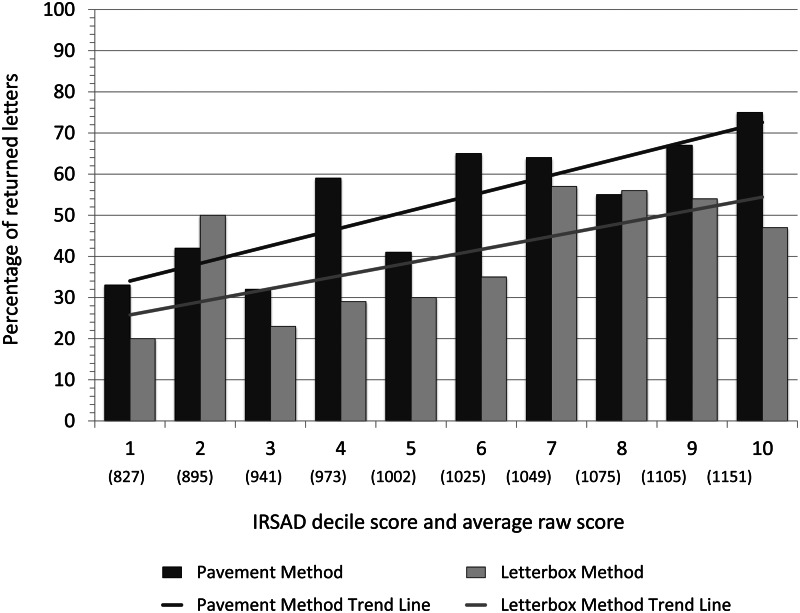
Association of socioeconomic advantage with altruism. The percentage of returned letters for the pavement and letterbox methods by the decile mid-point of the IRSAD (Index of Relative Socio-economic Advantage and Disadvantage) score of the SA1 area in which they were distributed. Note: the average raw score (in parentheses) represents the mid-point of the decile; it was calculated by averaging all raw scores that fell within each decile.

A total of 302 letters (50% of the number distributed) were returned from both the pavement and letterbox methods (Table 1; Table S3). The full IRSAD model was significantly different from the null model (*χ*^2^ = 772.4, *p* < 0.001). Table 2 and Figure 1 show that letters distributed in areas with higher IRSAD scores were significantly more likely to be returned than letters distributed in areas with lower IRSAD scores. The likelihood of a letter being returned also depended on the method used, with pavement letters being significantly more likely to be returned than letterbox letters (Table 2; Figure 1). Crime rate and the number of post boxes were not significantly related to letter return rates (Table 2). The variance of the random effect (suburb), i.e. the contribution of the random effect to the variation in the response variable, was 0.094 (SD = 0.306). Additional simplified models (without crime and/or post boxes) are presented in Tables S4–S6.

**Table 1.** The number (and percentage) of returned and not returned letters as a function of their distribution method

Returned	Pavement	Letterbox	Total
Yes	175 (58.3%)	127 (42.3%)	302 (50.3%)
No	125 (41.7%)	173 (57.7%)	298 (49.7%)
Total	300	300	600

**Table 2.** Generalized linear mixed model (GLMM) output on whether a letter was returned or not showing fixed effects for crime, letter distribution method, Index of Relative Socio-economic Advantage and Disadvantage (IRSAD) and number of post boxes. AIC: 784.4

Fixed effects	Estimate	SE	2.5% CI	97.5% CI	*p*
(Intercept)	−0.4862	1.1413	−0.7631	−0.2092	
Crime	−0.1905	0.1334	−0.4519	0.0709	0.153
Method (*Pavement*)	0.8336	0.1744	0.4919	1.1753	<0.001
IRSAD	0.3384	0.1299	0.0839	0.5930	0.009
Post boxes	0.1787	0.1177	−0.0520	0.4094	0.129

**Figure 2.**
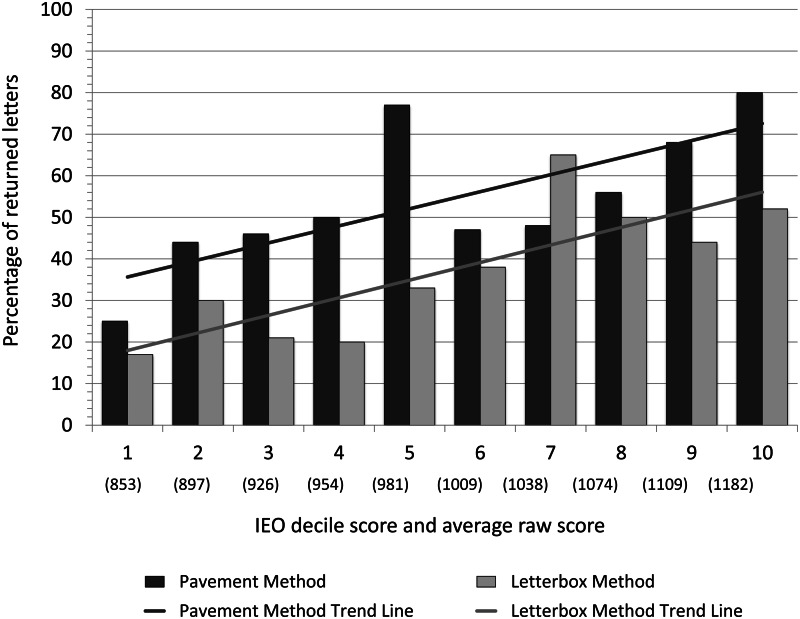
Association of education/occupation with altruism. The percentage of returned letters for the pavement and letterbox methods by the decile mid-point of the IEO (Index of Education and Occupation) score of the SA1 area in which they were distributed. Note: the average raw score in parentheses represents the mid-point of the decile; it was calculated by averaging all raw scores that fell within each decile.

**Figure 3.**
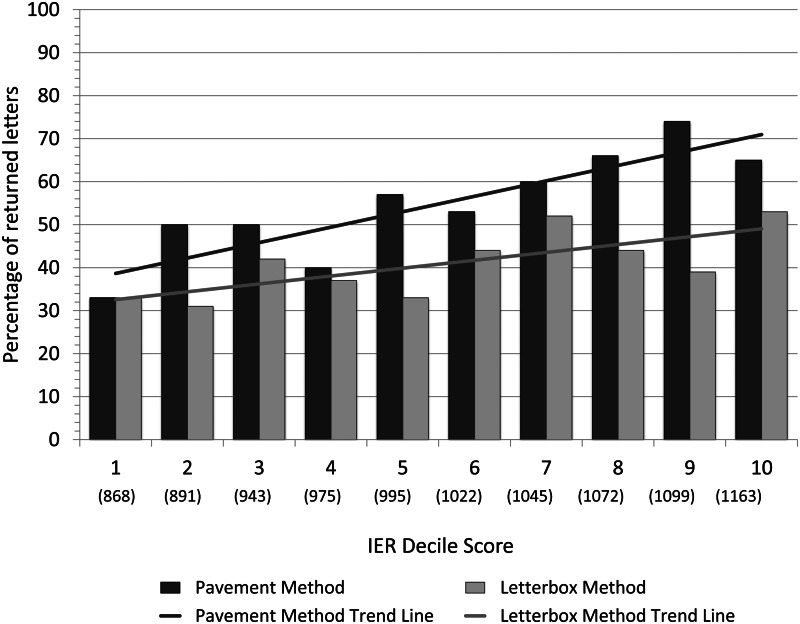
Association of economic resources with altruism. The percentage of returned letters for the pavement and letterbox methods by the decile mid-point of the IER (Index of Economic Resources) score of the SA1 area in which they were distributed. Note: the average raw score in parentheses represents the mid-point of the decile; it was calculated by averaging all raw scores that fell within each decile.

A follow-up GLMM with either IER or IEO was run (Table 3). Both models explained variation in the occurrence of prosocial behaviour associated with picking up a letter better than the respective null models (IER. *χ*^2^ = 777.6, *p* < 0.001; IEO. *χ*^2^ 765.5, *p* < 0.001). IEO was a significant predictor of whether a letter would be returned or not (Figure 2), but IER was not (Figure 3). The variance of the random effect (suburb) was 0.185 (SD = 0.430). The model with IEO also featured a lower AIC value (786.8) than both the model with IER (798.6) and the model with IRSAD (793.6), indicating that the IEO model was a better fit to the data. The distribution method was again significant for both models, with pavement letters more likely to be returned than letterbox letters. The number of post boxes did not influence letter return rates. Crime was not a significant predictor of letter return rates in the IEO model, but was significant in the IER model (Table 3). The variance of the random effect (suburb) was 0.041 (SD = 0.202). Additional simplified models (without crime and/or post boxes) are presented in Tables S7–S12.

**Table 3.** GLMMs outputs with either Index of Economic Resources (IER) or Index of Education and Occupation (IEO) as main fixed effects and letter return as a binary response term. AIC for IER model, 789.6; AIC for IEO model, 777.5

Model	Variables	Estimate	SE	2.5% CI	97.5% CI	*p*
IER	(Intercept)	−0.4838	0.1568	−0.7901	−0.1766	
	Crime	−0.3401	0.1448	−0.6240	−0.0563	0.019
	Method (*Pavement*)	0.8356	0.1745	0.4935	1.1777	<0.001
	IER	0.0835	0.1229	−0.1575	0.3245	0.497
	Post boxes	0.2607	0.1341	−0.0021	0.5236	0.052
IEO	(Intercept)	−0.4906	1.1318	−0.7490	−0.2321	
	Crime	−0.1412	0.1146	−0.3657	0.0834	0.218
	Method (*Pavement*)	0.8414	0.1748	0.4988	1.1839	<0.001
	IEO	0.4808	0.1181	0.2493	0.7123	<0.001
	Post boxes	0.0684	0.1102	−0.1476	0.2844	0.535

## Discussion

The current study revealed substantial and systematic variation in altruistic tendencies across urban suburbs of different socio-economic characteristics. This variance appears to be conditioned by the education and occupation level of residents in the suburb, and was not consistently influenced by economic resources or crime rate.

### Socio-economic status

Both the original lost letter experiment and the novel, modified letterbox method provided support for the hypothesis that area-level SES (measured by IRSAD) was positively correlated with helping behaviour. This result is in consensus with previous research reporting a link between SES and letter return rates in the lost letter experiment (Brown and Reed 1982; Grueter *et al*. 2016; Holland *et al*. 2012; Nettle *et al*. 2011; Silva and Mace 2014). Multiple drivers underlying the lower levels of prosociality in low-SES areas are conceivable, for example time constraints resulting from the need to make ends meet (Holland *et al*. 2012; Lynam *et al*. 2000), lower sense of control over the environment (e.g. Gallo *et al*. 2005) or – more mundanely – higher tolerance of litter rates (see Khatib *et al*. 2007).

However, the above finding is in conflict with studies by Piff *et al*. (2010, 2012) who found upper-class individuals to be less prosocial and more unethical in measures such as willingness to cooperate with a game partner and attitudes on charitable donations. Côté *et al*. (2015) recently showed that higher-income individuals are not more selfish across the board but a tendency to be less generous emerges only under conditions of high economic inequality. Piff *et al*. (2012) suggested that – among others – this was because high-SES people have abundant resources to deal with the downstream costs of unethical behaviour (e.g. money for a speeding fine), while lower-SES individuals may need to be more careful as they incur greater relative consequences for social deviation. Piff *et al*. (2012) proposed that low-SES individuals have a greater interest in the wellbeing of others because it affects their ability to draw resources from them. Thus lower-class individuals’ willingness to engage in altruistic behaviour can be seen a function of economic interdependence. Relatedly, Amir *et al*. (2018) invoked an uncertainty management framework to account for the greater prosociality observed in economic games among economically deprived children. In this framework, cooperation with social partners and prosociality reflect the adaptive internalization of a risk-mitigating strategy in the face of uncertain returns associated with early life deprivation.

The difference between the results of Piff *et al*. (2010, 2012) and lost letter-based studies could stem from the fact that the former analysed variation at the individual level, whereas the latter examined neighbourhood-level differences (Holland *et al*. 2012). Perhaps high-SES neighbourhoods foster altruism, yet within any one neighbourhood, the poorer individuals are more altruistic than the wealthier ones (Holland *et al*. 2012).

Another reason for the difference between our findings and those of Piff *et al*. (2010, 2012) could be that their experiments measured altruistic tendencies towards people in general (no specific group) in a range of environments. In contrast, the lost letter experiment used in this study measured altruistic behaviours within one's own ‘home environment’ (their suburb or street) towards (presumably) members of their own group; individuals who encountered lost letters would have probably assumed that the letter was distributed by a resident when walking through the area.

Lastly, as suggested by Holland *et al*. (2012), the experiments used to analyse altruism by Piff *et al*. (2010, 2012) may be more competitive than the small, cooperative task of returning a lost letter, resulting in different behaviours. For example, upper-class individuals may be more likely than lower-class individuals to return a letter in a cooperative task, but they may also be more likely to deceive another player in a laboratory-based economic game. Future studies should incorporate multiple measures of altruistic behaviour (such as those used by Piff *et al*. 2010, 2012) to determine if the patterns seen in this study are unique to the lost letter experiment.

### Socio-economic variables

The principal aim of this study was to disentangle the association of different socio-economic variables with altruistic behaviour. Crime was predicted to reduce altruism by lowering trust, but a suburb's crime rate was largely unrelated to the expression of prosocial behaviour. Only in the model where economic resources were included did crime rate become significant. Therefore, the variance explained by crime rate may be accounted for by other SES characteristics such as education, which was included in all other models. It may also be that crime has a threshold effect and needs to be at a certain rate before it begins to affect peoples’ altruistic tendencies. The suburbs analysed in this study may not have had sufficient crime rates to demonstrate this effect.

Economic resources, as a characteristic of SES, also did not have a significant effect on letter return rate. This suggests that demographic factors such as individuals’ assets, house prices and average household income are not related to suburb-level altruistic behaviours. Holland *et al*. (2012) suggested that low-SES individuals may be too preoccupied with meeting their individual needs to be willing to spend time helping others. This hypothesis suggests that individuals with more economic resources will be better equipped to meet their needs and will, therefore, have more time and energy to engage altruistically with others. The current dataset does not rule out the hypothesis that, when time itself is not a limited resource, people may be more willing to engage in prosocial behaviours. It should be noted that the location of this study does not experience widespread socio-economic deprivation where a great proportion of individuals do not have access to basic needs such as clean water, food and housing. Perhaps this hypothesis may be relevant in more economically deprived contexts where economic resources may influence altruism.

IEO was found to be significantly associated with whether a letter would be returned or not. The effect of IRSAD on letter return rate may largely be explained by the composite variables that it shares with IEO. This finding suggests that the component of SES that affects a neighbourhood's letter return rate is the education and occupation status of individuals in that suburb. To our knowledge this is a novel finding that has not been reported previously. However, along a similar vein, there is one recent study which documented a positive correlation between historical rates of primary education and civic honesty (Cohn *et al*. 2019). Because IEO incorporates both education and occupation variables, we cannot distinguish whether both, or just one or the other, of these variables influence altruistic behaviour within a suburb.

This study has isolated education and occupation as the likely leading socio-economic variables behind the often found relationship between SES and letter return rates. However, we still do not fully understand the mechanism behind this link. We do not know what aspects of education and occupation status may lead individuals within a suburb to behave more altruistically. Education and occupation may also be associated with a third variable that may be driving the patterns in the results. For example, individuals who have achieved a high education level or who are in high-status jobs are more likely to possess greater cognitive abilities (Schmidt and Hunter 2004; Strenze 2007). It may thus be possible that the significant effect of IEO on letter return rate reflects an underlying effect of cognitive ability. Previous studies in behavioural economics have shown a link between cognitive ability and altruistic behaviour (Jones 2008). Cognitive ability has been found to be negatively correlated with a preference for immediate rewards and impulsivity (Jensen 1998; de Wit *et al*. 2007). Cognition in more stressful and harsh environments associated with lower SES may be focused more on temporal discounting and lower levels of self-control (Coall *et al*. 2012; Frankenhuis *et al*. 2016; Mullainathan and Shafir 2013; Sheehy-Skeffington and Rea 2017), conditions that discourage altruistic behaviour (Osiński *et al*. 2017). It is important to note that extrapolating from SES at a relatively crude area-level analysis to individual differences in cognitive ability (and thus altruism) is problematic. The relationship between these factors and education is probably more complex, and dependent on many factors (e.g. opportunity, value placed on education, etc.).

An evolutionary mechanism underlying the finding that education and occupation are the primary drivers of prosocial behaviour may be that educated people have more opportunities to learn, to be taught and to receive feedback and thus are more likely to adopt or maintain cultural norms of prosociality. Individual behavioural decisions (as to whether to act proscocially) are influenced by expectations of the behaviour of others in the local social environment (Bichierri and Xiao 2009). In turn, these decisions also influence the local social environment, by conveying to others information about local norms of cooperative behaviour (cf. Schroeder *et al*. 2014).

### Modified lost-letter experiment

The novel letterbox method incorporated in this study featured a significantly lower return rate compared with the original pavement method. Both methods, however, exhibited the same SES patterns in the data. One explanation for the differing return rates is that ignoring a letterbox letter ends all future possibilities for the letter to be returned, but pavement letters may be picked up by someone else (however, one could also argue that receiving a lost letter in someone's letterbox increases the recipient's pressure to do something about it). Another possible explanation is that returning a pavement letter may incur a smaller cost in terms of time and effort, because an individual could already be heading in the direction of a post box, compared who a letterbox recipient who would have to make a separate trip. Furthermore, individuals may behave more prosocially when encountering pavement letters because there is a chance that their actions are being observed by bystanders and influence their reputation (*sensu* Raihani and Bshary, 2015). Additionally, there remains the possibility that letterbox recipients may have uncertainty about what to do with the wrongly addressed letter.

The similar socio-economic patterns found in the results from both methods suggest that the letterbox method may be a useful alternative to the pavement method as it may not be as susceptible to some of the potentially confounding variables such as non-residents encountering the letters and differing rates of pedestrian foot traffic in different neighbourhoods. The letterbox method has additional advantages that should be considered for future experiments. The method enables the letter to be distributed at any time, unlike pavement letters, which must be distributed on wind- and rain-free evenings. The letterbox method also eliminates the lengthy process of distributing letters by hand and provides easy access to remote or rural areas. Additionally, the letterbox method allows for more letters to be distributed in any given area, owing to the elimination of the possibility of individuals encountering multiple letters while walking through a neighbourhood.

### Cultural group selection and prosociality

Cultural group selection theory posits that groups whose members engage altruistically with each other are more successful in intergroup competition than groups whose members lack such locally stable cooperative cultural norms (Henrich 2004; Richerson *et al*. 2016). However, the great variation in altruistic tendencies exhibited by the different suburbs suggests that cultural group selection does not function at the scale of the city. Instead, we may see large populations splitting up into smaller sub-groups with their own set of altruistic norms which may be the result of cultural group selection operating on this smaller scale. However, since populations of city suburbs are not natural groups but administrative divisions, it is unclear if these are subject to cultural group selection. Alternatively, variation in altruism attributed to different cultural norms could in fact reflect individual adaptations to different *environments* with varying levels of socio-economic harshness (Mace and Silva 2016).

## Conclusions

This study has provided new insight into human altruistic behaviour and has reinforced that altruistic behaviour is contingent on contextual factors. Specifically, between-suburb variation in altruism was found to co-vary with SES. Education and occupation were the components of SES found to increase the prevalence of unconditional altruism within a suburb. This relationship may be due to differences in individual cognitive ability and self-control and the possibility that education positively correlates with an individual's ability or willingness to adhere to local norms of prosociality. Our study also offers a new variant of the lost letter experiment, the letterbox method, which is advantageous in reducing confounds and easing the logistics of fieldwork. Knowledge about the manifestation of human altruism and community norms across gradients of SES could also have practical implications for charitable organizations and local government policy.

## Data Availability

The data used in this study are available as Supplementary Files.

## Supplementary material

For supplementary material accompanying this paper visit http://dx.doi.org/10.1017/ehs.2019.16. click here to view supplementary material
