# Supplementary material for: Educational attainment is associated with unconditional helping behaviour
Source: Evol Hum Sci. 2019 Dec 11;1:e15. doi: 10.1017/ehs.2019.16 (PMC10427308; doi:10.1017/ehs.2019.16)
Supplement: Supplementary file 1 [file S2513843X19000161sup001.docx]

**Supplementary Information / Appendices**

**Table S1.** Socioeconomic information for the suburbs in which the lost letter experiment was conducted

| Suburb name | Number of postboxes | Suburb IRSAD | | Suburb IER | | Suburb IEO | | SA1 IRSAD (mean and range) | SA1 IER (mean and range) | SA1 IEO (mean and range) | Crime rate |
| --- | --- | --- | --- | --- | --- | --- | --- | --- | --- | --- | --- |
|  |  | Raw value | Decile value | Raw value | Decile value | Raw value | Decile value |  |  |  |  |
| Alfred Cove | 1 | 1105 | 10 | 1075 | 9 | 1120 | 10 | 1107 (1082–1131) | 1077 (1051–1098) | 1123 (1080–1180) | 1726 |
| Beechboro | 3 | 990 | 5 | 1044 | 7 | 933 | 3 | 961 (914–1004) | 1005 (950–1068) | 921 (897–967) | 3174 |
| Bicton | 2 | 1086 | 9 | 1049 | 7 | 1115 | 10 | 1093 (993–1159) | 1054 (940–1140) | 1119 (1044–1156) | 1970 |
| Clarkson | 2 | 1012 | 6 | 1033 | 6 | 972 | 5 | 1004 (946–1095) | 1033 (972–1124) | 960 (863–1042) | 4644 |
| Coolbinia | 1 | 1127 | 10 | 1124 | 10 | 1131 | 10 | 1127 (1117–1171) | 1122 (1077–1170) | 1129 (1096–1170) | 3325 |
| Cottesloe | 9 | 1144 | 10 | 1078 | 9 | 1195 | 10 | 1147 (1087–1188) | 1087 (959–1148) | 1195 (1156–1224) | 1840 |
| Craigie | 1 | 1016 | 6 | 1028 | 6 | 971 | 5 | 1010 (967–1066) | 1024 (968–1077) | 969 (913–1035) | 2754 |
| East Victoria Park | 6 | 1045 | 8 | 987 | 4 | 1094 | 9 | 1048 (990–1101) | 996 (879–1078) | 1096 (1052–1141) | 5777 |
| Ferndale | 2 | 1021 | 7 | 1022 | 6 | 998 | 6 | 1018 (968–1072) | 1021 (990–1099) | 996 (946–1025) | 2044 |
| Hamilton Hill | 6 | 951 | 3 | 945 | 2 | 980 | 5 | 963 (870–1003) | 966 (893–1019) | 981 (905–1040) | 4978 |
| Iluka | 1 | 1162 | 10 | 1194 | 10 | 1089 | 9 | 1160 (1135–1174) | 1190 (1168–1220) | 1086 (1041–1125) | 1096 |
| Koondoola | 1 | 856 | 1 | 914 | 2 | 867 | 1 | 854 (808–931) | 912 (872–991) | 868 (829–905) | 4366 |
| Marangaroo | 1 | 974 | 4 | 1032 | 6 | 942 | 4 | 976 (918–1026) | 1032 (969–1089) | 943 (902–1003) | 2470 |
| Merriwa | 2 | 937 | 3 | 1000 | 5 | 891 | 2 | 952 (890–1035) | 1008 (967–1104) | 901 (854–945) | 2888 |
| Palmyra | 6 | 1036 | 7 | 997 | 4 | 1073 | 9 | 1041 (925–1075) | 1006 (845–1043) | 1075 (1051–1097) | 2134 |
| Peppermint Grove | 1 | 1155 | 10 | 1108 | 10 | 1196 | 10 | 1158 (1123–1186) | 1114 (1046–1145) | 1191 (1171–1207) | 2298 |
| Riverton | 2 | 1058 | 8 | 1031 | 6 | 1066 | 9 | 1061 (1031–1082) | 1039 (991–1089) | 1067 (1034–1090) | 1460 |
| Scarborough | 7 | 1076 | 9 | 1007 | 5 | 1098 | 10 | 1079 (1027–1140) | 1021 (940–1098) | 1104 (1075–1148) | 2854 |
| Shenton Park | 3 | 1107 | 10 | 1032 | 6 | 1188 | 10 | 1122 (1010–1153) | 1055 (908–1097) | 1192 (1148–1219) | 1402 |
| Wilson | 2 | 1027 | 7 | 976 | 4 | 1062 | 9 | 1035 (934–1122) | 987 (883–1098) | 1062 (997–1142) | 2813 |

IRSAD is the Index of Relative Socio-economic Advantage and Disadvantage; IER is the Index of Economic Resources; IEO is the Index of Education and Occupation (see main text for details). These measures were obtained for each suburb, and also smaller areas within each suburb called Statistical Areas Level 1 (SA1). Crime rate is number of events per 100,000 people.

**Table S2**. Model output from GLMMs on whether a letter was returned or not showing the following fixed effects: crime, IRSAD, number of post boxes and letter distribution method. For the first GLMM, suburb-level data were uses, for the second one SA1-level data

| Model | Variables | Estimate | SE | *p* |
| --- | --- | --- | --- | --- |
| Suburb IRSAD | (Intercept) | −5.7544 | 1.8127 |  |
|  | Crime | −0.0001 | 0.0001 | 0.277 |
|  | Method (*Pavement*) | 0.6973 | 0.1726 | 0.001 |
|  | Suburb IRSAD | 0.0053 | 0.0016 | 0.001 |
|  | Post boxes | 0.0570 | 0.0466 | 0.222 |
| SA1 IRSAD | (Intercept) | −4.103 | 1.6120 |  |
|  | Crime | −0.0002 | 0.0001 | 0.110 |
|  | Method (pavement) | 0.6933 | 0.1722 | 0.001 |
|  | SA1 IRSAD | 0.0038 | 0.0014 | 0.006 |
|  | Post boxes | 0.0642 | 0.0468 | 0.170 |

**Table S3.** Summary data by suburb with total number of returned letters using the two distribution methods (letterbox vs. pavement; see main text for details)

| Suburb name | Number of letters returned | | |
| --- | --- | --- | --- |
|  | Letterbox | Pavement | Total |
| Alfred Cove | 7 | 10 | 17 |
| Beechboro | 3 | 6 | 9 |
| Bicton | 6 | 11 | 17 |
| Clarkson | 3 | 6 | 9 |
| Coolbinia | 8 | 8 | 16 |
| Cottesloe | 6 | 15 | 21 |
| Craigie | 6 | 8 | 14 |
| East Victoria Park | 6 | 11 | 17 |
| Ferndale | 9 | 11 | 20 |
| Hamilton Hill | 3 | 7 | 10 |
| Iluka | 6 | 9 | 15 |
| Koondoola | 3 | 5 | 8 |
| Marangaroo | 3 | 6 | 9 |
| Merriwa | 3 | 4 | 7 |
| Palmyra | 11 | 12 | 23 |
| Peppermint Grove | 7 | 14 | 21 |
| Riverton | 7 | 6 | 13 |
| Scarborough | 6 | 5 | 11 |
| Shenton Park | 10 | 11 | 21 |
| Wilson | 4 | 9 | 13 |

**Table S4.** GLMM output on whether a letter was returned or not showing fixed effects for crime, letter distribution method, IRSAD and crime. Post boxes were not included in this model

| Fixed effects | Estimate | SE | *p* |
| --- | --- | --- | --- |
| (Intercept) | −0.487 | 0.145 |  |
| Crime | −0.134 | 0.132 | 0.312 |
| Method (*Pavement*) | 0.833 | 0.174 | <0.001 |
| IRSAD | 0.375 | 0.131 | 0.004 |

Random effect (suburb) variance = 0.116; SD = 0.340.

**Table S5.** GLMM output on whether a letter was returned or not showing fixed effects for crime, letter distribution method, IRSAD and post boxes. Crime was not included in this model

| Fixed effects | Estimate | SE | *p* |
| --- | --- | --- | --- |
| (Intercept) | −0.486 | 0.143 |  |
| Post boxes | 0.128 | 0.114 | 0.262 |
| Method (*Pavement*) | 0.832 | 0.174 | <0.001 |
| IRSAD | 0.430 | 0.114 | <0.001 |

Random effect (suburb) variance = 0.104; SD = 0.322.

**Table S6.** GLMM output on whether a letter was returned or not showing fixed effects for crime, letter distribution method and IRSAD. Post boxes and crime were not included in this model

| Fixed effects | Estimate | SE | *p* |
| --- | --- | --- | --- |
| (Intercept) | −0.486 | 0.145 |  |
| Method (*Pavement*) | 0.832 | 0.174 | <0.001 |
| IRSAD | 0.437 | 0.116 | <0.001 |

Random effect (suburb) variance = 0.118; SD = 0.344.

**Table S7.** GLMM output on whether a letter was returned or not showing fixed effects for crime, letter distribution method, IER and crime. Post boxes were not included in this model

| Fixed effects | Estimate | SE | *p* |
| --- | --- | --- | --- |
| (Intercept) | −0.486 | 0.167 |  |
| Crime | −0.298 | 0.154 | 0.054 |
| Method (*Pavement*) | 0.836 | 0.175 | <0.001 |
| IER | 0.057 | 0.126 | 0.649 |

Random effect (suburb) variance = 0.251; SD = 0.501.

**Table S8.** GLMM output on whether a letter was returned or not showing fixed effects for crime, letter distribution method, IER and post boxes. Crime was not included in this model

| Fixed effects | Estimate | SE | *p* |
| --- | --- | --- | --- |
| (Intercept) | −0.483 | 0.168 |  |
| Post boxes | 0.203 | 0.45 | 0.162 |
| Method (*Pavement*) | 0.833 | 0.174 | <0.001 |
| IER | 0.172 | 0.122 | 0.160 |

Random effect (suburb) variance = 0.258; SD = 0.508.

**Table S9.** GLMM output on whether a letter was returned or not showing fixed effects for crime, letter distribution method and IER. Crime and post boxes were not included in this model

| Fixed effects | Estimate | SE | *p* |
| --- | --- | --- | --- |
| (Intercept) | −0.484 | 0.175 |  |
| Method (*Pavement*) | 0.834 | 0.174 | <0.001 |
| IER | 0.138 | 0.123 | 0.259 |

Random effect (suburb) variance = 0.308; SD = 0.555.

**Table S10.** GLMM output on whether a letter was returned or not showing fixed effects for crime, letter distribution method, IEO and crime. Post boxes were not included in this model

| Fixed effects | Estimate | SE | *p* |
| --- | --- | --- | --- |
| (Intercept) | −0.491 | 0.132 |  |
| Crime | −0.115 | 0.107 | 0.283 |
| Method (*Pavement*) | 0.841 | 0.175 | <0.001 |
| IEO | 0.510 | 0.109 | <0.001 |

Random effect (suburb) variance = 0.043; SD = 0.207.

**Table S11.** GLMM output on whether a letter was returned or not showing fixed effects for crime, letter distribution method, IEO and post boxes. Crime was not included in this model

| Fixed effects | Estimate | SE | *p* |
| --- | --- | --- | --- |
| (Intercept) | −0.491 | 0.134 |  |
| Post boxes | 0.018 | 0.105 | 0.864 |
| Method (*Pavement*) | 0.841 | 0.175 | <0.001 |
| IEO | 0.550 | 0.106 | <0.001 |

Random effect (suburb) variance = 0.051; SD = 0.225.

**Table S12.** GLMM output on whether a letter was returned or not showing fixed effects for crime, letter distribution method, IEO. Post boxes and crime were not included in this model

| Fixed effects | Estimate | SE | *p* |
| --- | --- | --- | --- |
| (Intercept) | −0.491 | 0.134 |  |
| Method (*Pavement*) | 0.841 | 0.175 | <0.001 |
| IEO | 0.555 | 0.102 | <0.001 |

Random effect (suburb) variance = 0.051; SD = 0.225.
